# Assessment of tumor promoting effects of amniotic and umbilical cord mesenchymal stem cells in vitro and in vivo

**DOI:** 10.1007/s00432-019-02859-6

**Published:** 2019-02-25

**Authors:** Ming-Yao Meng, Lin Li, Wen-Ju Wang, Fei-Fei Liu, Jian Song, Song-Lin Yang, Jing Tan, Hui Gao, Yi-Yi Zhao, Wei-Wei Tang, Rui Han, Kai Zhu, Li-Wei Liao, Zong-Liu Hou

**Affiliations:** 1 Central Laboratory of Yan’an Hospital Affiliated to Kunming Medical University, No. 245 East of Renmin Road, Kunming, 650051 Yunnan People’s Republic of China; 2 Key Laboratory of Tumor Immunological Prevention and Treatment of Yunnan Province, Kunming, Yunnan People’s Republic of China; 3 Yunnan Cell Biology and Clinical Translation Research Center, Kunming, Yunnan People’s Republic of China; 4 Kunming Medical University, Kunming, Yunnan People’s Republic of China

**Keywords:** Amniotic mesenchymal stem cells, Umbilical cord mesenchymal stem cells, MSCs, Cancer, Cytokines

## Abstract

**Purpose:**

Human mesenchymal stem cells (hMSCs) have been applied in a variety of therapies recently. However, the role of MSCs in tumor progression remains largely elusive. Some studies demonstrated that MSCs can promote tumor growth, while others had opposite results. Therefore, the lack of evidence about the effect of MSCs on tumor cells impedes its further use.

**Methods:**

In the current study, hMSCs from amniotic membrane (hAMSCs) and umbilical cord (hUCMSCs) were used to evaluate the effects of MSCs on tumor development in vitro and in vivo. Two different animal models based on subcutaneous xenograft bearing nude mice and a murine experimental metastatic model were established for in vivo study. Moreover, cytokines regulated by MSCs co-cultured with cancer cells SPC-A-1 were also analyzed by cytokine array.

**Results:**

Our results indicated that hUCMSCs not only did not promote proliferation in cancer cells, but also inhibited migration. In addition, they inhibited tube formation in human umbilical vein endothelial cells (HUVECs). Although hAMSCs also showed inhibitory effects on cancer cell motility, the proliferation of cancer cells was indeed enhanced. The in vivo data revealed that hUCMSCs did not promote tumor progression in lung adenocarcinoma and gastric carcinoma xenografts. Nevertheless, hAMSCs could do. The results from murine experimental metastatic model also demonstrated that neither hUCMSCs nor hAMSCs significantly enhanced the lung metastasis. The data from cytokine array showed that 11 inflammatory factors, 8 growth factors and 11 chemokines were remarkably secreted and changed.

**Conclusions:**

In view of the data from in vitro and in vivo studies, the exploitation of hUCMSCs in new therapeutic strategies should be safe compared to hAMSCs under malignant conditions. Moreover, this is the first report to systematically elucidate the possible molecular mechanisms involved in UCMSC- and AMSC-affected tumor growth and metastasis.

**Electronic supplementary material:**

The online version of this article (10.1007/s00432-019-02859-6) contains supplementary material, which is available to authorized users.

## Introduction

Mesenchymal stem cells (MSCs) are a promising tool in cell therapies due to their multipotent, self-renewal, and immunomodulatory properties (Pittenger et al. 1999). MSCs can differentiate into multiple cell types such as osteoblasts, adipocytes, chondrocytes, myocytes, fibroblasts, epithelial cells and neurons. Therefore, MSCs show considerable therapeutic potential in genetic and acquired human diseases associated with the loss of specific tissues such as cardiovascular disease, diabetes, Alzheimer’s disease, multiple sclerosis, etc. (Arutyunyan et al. 2016; Uccelli et al. 2008). Several studies reported that MSCs could migrate to the sites of tumors. This migratory ability of MSCs might enable them to be utilized as the delivery vehicle for other anti-cancer drugs. However, the therapeutic application of MSCs in human malignancies could only be carried out after the validation of the effects of MSCs themselves (Serakinci et al. 2014). Currently, there are only limited studies investigating the properties of MSCs by animal models revealing inconsistent results. Some studies showed MSCs promoted tumor growth and metastasis, while others had opposite results. The controversial results and poorly understood mechanisms presented a serious obstacle to using MSCs clinically. Therefore, more studies about the effects of MSCs on tumor cells are needed to provide solid scientific evidences. Since 2006, our group has established stable MSC lines from human amniotic and umbilical cord mesenchymes (Hou et al. 2013; Meng et al. 2012). Among different MSCs, human amniotic mesenchymal stem cells (hAMSCs) and human umbilical cord mesenchymal stem cells (hUCMSCs) are the most potent and attractive stem cell sources for clinical use due to their easy, painless and safe (low risk of viral infection) collection procedures. Therefore, hAMSCs and hUCMSCs would be employed for our present study.

To further explore the role of MSCs on tumors, the effects of hAMSCs and hUCMSCs on lung adenocarcinoma cells, gastric carcinoma cells and human umbilical vein endothelial cells (HUVEC) were evaluated in vitro and in vivo. In addition, the possible molecular mechanisms involved in MSC-affected tumor growth and metastasis were also explored by the comprehensive cytokine secretion profile.

## Materials and methods

### Isolation, culture and phenotyping of hAMSCs and hUCMSCs

Human umbilical cords were obtained from mothers who had given birth at Yan’an Hospital of Kunming Medical University. All subjects had obtained written informed consent before the study, which was approved by the ethics committee of Yan’an Hospital of Kunming Medical University. The protocol of the study conformed to the ethical guidelines of the 1989 Declaration of Helsinki. The hAMSCs and hUCMSCs were isolated and amplified according to our previous study (Meng et al. 2012). The morphology of cells was observed and the photos were taken by a phase contrast microscope (Olympus, Tokyo, Japan). The surface markers of hAMSCs and hUCMSCs including CD73, CD29, CD14, CD166, CD117, CD49, CD90, CD44, HLA-DR, CD45, CD34 were analyzed by flow cytometry (FACScalibur; Becton Dickinson) and CellQuest software (Becton Dickinson). The antibodies against the above surface markers were purchased from BD Biosciences, USA.

### Multi-differentiation capabilities of hAMSCs and hUCMSCs

The pluripotency of hAMSCs and hUCMSCs were validated. Adipogenic, osteogenic and neurogenic differentiation experiments were performed as described previously (Meng et al. 2012). In brief, hAMSCs and hUCMSCs were cultured in Minimum Essential Medium Eagle—Alpha Modification (α-MEM) for 24 h. For adipogenic differentiation, the medium was then changed to Dulbecco’s modified Eagle’s medium (DMEM) supplemented with 10% fetal bovine serum (FBS), 100 nmol/L dexamethasone, 10 mmol/L glycerophosphate and 0.2 mmol/L ascorbic acid 2-phosphate for 3 weeks and analyzed by Oil Red O staining. For osteogenic differentiation, α-MEM were changed to DMEM with low glucose (DMEM-LG) supplemented with 10% FBS, 100 nmol/L dexamethasone, 10 mmol/L-glycerophosphate, and 0.2 mmol/L l-ascorbic acid (Sigma-Aldrich) for about 3 weeks and assayed by von Kossa staining. The chondrogenic differentiation was conducted using the MSC go Chondrogenic XF™ Kit (Biological Industries, USA) for 3 weeks. The cells were then fixed with 10% formaldehyde for 24 h and embedded in paraffin. The blocks were sectioned at 4 µm thickness and stained with Alcian Blue Staining Kit (ScienCell, Carlsbad, CA Chondrogenesis Protocol: USA) according to the manufacturer’s instruction. The morphologies of cartilage lacuna and sulfated proteoglycan were identified under a light microscope (Primo Vert, Carl Zeiss Microscopy GmbH, Germany). For neural differentiation, the cells were cultured in α-MEM supplemented with 2% (v/v) FBS, 10 ng/mL basic fibroblast growth factor, 10 ng/mL platelet-derived growth factor, 0.1 µM dexamethasone, 0.5 µM linoleic acid and 50 mg/mL gentamicin sulfate (all from Sigma-Aldrich, USA) for 3 weeks and analyzed by immunofluorescence microscopy after incubation with human monoclonal antibodies against neurofilament M (1:200; Millipore, Temecula, CA, USA).

### Culturing of other cell lines

Human pulmonary adenocarcinoma cell line SPC-A-1 and gastric carcinoma cell line BGC-823 were purchased from Cell Bank of Shanghai Institutes for Biological Sciences, Chinese Academy of Sciences, China. Human diploid cell line KMB-17, which originated from embryonic lung fibroblasts, was obtained from Institute of Medical Biology, Chinese Academy of Medical Sciences and Peking Union Medical College, Kunming, China.

SPC-A-1, BGC-823 and KMB-17 cells were maintained in RPMI 1640 medium (Hyclone Laboratories) supplemented with 10% (v/v) FBS, 100 IU/mL penicillin and 100 mg/mL streptomycin, and incubated at 37 °C in a 5% CO_2_, 95% humidified atmosphere.

### Harvest of hAMSC- and hUCMSC-conditioned medium

The hAMSCs and hUCMSCs at passage 5 were cultured in 175 cm^2^ flask in 50 mL α-MEM supplemented with 10% FBS, 100 IU/mL penicillin and 100 mg/mL streptomycin until cells were approximately 80–90% confluent. At that time the medium was replaced with serum-free DMEM/F12 for 48 h. Subsequently, the conditioned medium was collected and centrifuged at 1000 rpm for 5 min. Then, it was filtered through a 0.22 µm syringe filter and conserved as AMSC-conditioned medium (AMSC-CM) and UCMSC-conditioned medium (UCMSC-CM) at -80 °C until use. The medium from KMB-17 was used as the control comparing with MSC-CM in our experiments. The procedure for collecting KMB-17-conditioned medium (KMB-CM) was the same as that of the MSC-CM.

### Cell proliferation by CCK-8 Assay

SPC-A-1 and BGC-823 cells (both at 5 × 10^3^ cells/well) were seeded in 96-well microplates for overnight. AMSC-CM or UCMSC-CM was added into cancer cells culture medium (RPMI 1640 medium with 10% FBS (v/v), 100 IU/mL penicillin and 100 mg/mL streptomycin) with concentrations of 20%, 40% and 60% respectively. After 72 h incubation, WST-8 (CCK-8, Dojindo Cell Counting Kit-8, Japan) was added to each well for the detection of cell proliferation according to the manufacturer’s instruction. The samples were firstly incubated at 37 °C for 1.5–2 h. The absorbance values at 450 nm were then measured by a microplate spectrophotometer (Varioskan Lux, Thermo Scientific, USA). Absorbance readings were blanked against the medium alone and the cell viability was expressed relative to vehicle control data.

### Cell migration assay

The motility of SPC-A-1 and BGC-823 cells incubated with UCMSC-CM or AMSC-CM was assessed by the scratch wound assay. SPC-A-1 cells (5.0 × 10^5^ cells/well) and BGC-823 cells (4.5 × 10^5^ cells/well) were seeded in the Culture-Insert 2 Well in µ-Dish^35mm, low^ (ibidi GmbH, Martinsried, Germany) and allowed for attachment for 24 h. Then, the Culture-Insert 2 Well was removed to create the cell-free gap. The medium was changed to UCMSC-CM or AMSC-CM for 12, 24, 36, or 48 h and each well was photographed at 40 × magnification under a light microscope. The percentages of open wound area were measured and calculated using the Image J 8.0 software. The motility was determined by the decrease in closed wound area as compared with control.

### Matrigel invasion assay

The migration abilities of SPC-A-1 or BGC-823 cells were evaluated by the Matrigel invasion assay. Briefly, SPC-A-1 or BGC-823 cell suspension (4 × 10^4^ cells in 100 µl UCMSC-CM or AMSC-CM) was added into each transwell filter upper chamber (8 µm pore size, Corning, USA). The filters were pre-coated with 200 µg/mL Matrigel in advance. 500 µL of complete cell culture medium (RPMI 1640 medium with 10% FBS v/v), serving as chemoattractant medium, were added to the lower chamber. The cells were migrated to the lower chamber after incubation for 24 h at 37 °C. The cells on the bottom surface of filter membrane were fixed with methanol and stained with crystal violet staining solution. Four areas of each stained filter were randomly photographed at 100x magnification under a light microscope. The migrated cancer cells were quantified by manual counting. Three independent experiments were performed with duplicate wells each. The changes in number of migrated cells were expressed as a percentage of control values.

### Tube formation assay

To evaluate the effect of UCMSC-CM or AMSC-CM on formation of capillary tube-like structures, human umbilical vein endothelial cells (HUVEC) were used in Matrigel-based assay. Briefly, a 24-well plate coated with Matrigel (0.5 mL/well, BD Biosciences, USA) was incubated at 37 °C for 0.5 h for gel solidification. HUVEC at 7.5 × 10^4^ cells/well in 500 µl UCMSC-CM, AMSC-CM or DMEM/F12 (as control) media were added into separate wells and incubated for 8 h. UCMSC-CM and AMSC-CM was prepared from three independent experiments.

The enclosed networks of tubes were photographed under a light microscope. The total tube length of the tube structures in each photograph was analyzed by Image J 8.0 software.

### Cytokine array

A multitude of cytokines secreted from MSCs are known to confer multiple functions in cancer development such as pro- and anti-inflammation, proliferation, differentiation, chemoattraction, etc. (Feng and Chen 2009; Park et al. 2009; Serakinci et al. 2014). To assess the mechanisms involved in the regulatory effects of MSCs on cancer cells, the changes of cytokine level in the co-cultured medium were analysed. Briefly, hAMSCs or hUCMSCs (1 × 10^5^ cells/well) and SPC-A-1 (1 × 10^5^ cells/well) were co-cultured in 6-well plates. After 24 h, the medium was changed to a serum-free one (DMEM/F12 : RPMI 1640 = 1:1) and incubated for another 48 h. Subsequently, the medium was collected and stored at − 80 °C. The AMSC-CM, UCMSC-CM and RPMI 1640 medium (without serum) from SPC-A-1 alone were also collected separately. The production of cytokines including inflammatory factors, growth factors and chemokines were measured by a commercially available kit Quantibody Human Cytokine Antibody Array 2000 (RayBiotech, Inc., Norcross, GA, USA) according to the instruction of the manufacturer. The level of cytokines from the samples was calculated according to corresponding standards.

### In vivo evaluation of the cancer-promoting activity of MSCs

To evaluate the effect of hUCMSCs and hAMSCs on tumor formation and promotion in vivo, the murine subcutaneous xenograft cancer model was employed in the present study. The animal studies were reviewed and approved by the animal experimentation ethics committees of Yan’an Hospital of Kunming Medical University. Male BALB/c nude mice (6–8 wks of age) were provided by Hunan SJA Laboratory Animal Co., Ltd. The mice were bred and maintained in pathogen-free conditions. Numerous studies had previously reported that interleukin-6 (IL-6) was involved in the promotion of cancer growth (Tu et al. 2012). Therefore, one group of animals inoculated with cancer cells plus IL-6 was also included in our experiment. There were altogether 5 groups in the study: (1) SPC-A-1 or BGC-823 alone, (2) SPC-A-1 or BGC-823 with KMB-17, (3) SPC-A-1 or BGC-823 with hUCMSCs, (4) SPC-A-1 or BGC-823 with hAMSCs, (5) SPC-A-1 or BGC-823 with IL-6, with 6–7 mice allocated randomly into each group. Briefly, human cancer cells (SPC-A-1 or BGC-823, 1 × 10^6^ cells/mouse) were injected subcutaneously into the back of the mice alone or mixed with hUCMSCs, hAMSCs, KMB-17 (all of three using 1 × 10^6^ cells/mouse) or IL-6 (25 ng/mouse) on day 0. Tumor volume was measured every other day with a caliper and calculated using the formula (length × width^2^)/2 for 22 days. The mice were then euthanized and the tumors were dissected out. Tumor tissues were fixed in formaldehyde and processed for paraffin embedding. The paraffin-embedded tissue blocks were sectioned according to a previous study (Li et al. 2012), and were further used to assess the expression of Ki-67.

In another set of experiment, SPC-A-1 cells or BGC-823 cells (1 × 10^6^ cells/mouse) in PBS were injected subcutaneously into the back of the mice. After 12 days, the mice were randomized into four groups with 5–6 animals in each group. Treatment with hUCMSC or hAMSC (5 × 10^5^ cells/mouse), IL-6 (25 ng/mouse) or saline (as control) were conducted by i.v. injection through tail vein. Tumor volume was monitored every other day by caliper and calculated using the same formula with above. On day 22, the animals were killed and the tumors from the mice were collected for further analyses.

### In vivo evaluation of the cancer metastatic activity of MSCs

To further access the effect of hUCMSCs and hAMSCs on cancer metastasis in vivo, the murine experimental metastatic model was performed as previously described with modification (Li et al. 2018). SPC-A-1 cells or BGC-823 cells in PBS (1 × 10^6^ cells/mouse) were injected into male BALB/c nude mice via tail vein. After 24 h, the mice were randomized into four groups with 6–7 animals in each group. The hUCMSCs or hAMSCs (5 × 10^5^ cells/mouse), IL-6 (25 ng/mouse) or saline (as control) were also injected via tail vein. On day 33, the mice were killed by cervical dislocation. The liver, lungs and tibia were dissected out and fixed with 10% formalin. The tibia was decalcified for 3 days. Then, all of the samples were processed for paraffin embedding and sectioned. The sections of lungs, liver or bone were stained with haematoxylin & eosin (H&E), and the tumor lesions in these organs were calculated according to previous studies (Li et al. 2017; Luo et al. 2013). The tumor lesions in lungs, defined as the tumor area, was calculated from the sections and expressed as an average tumor area per group in absolute units (µm^2^).

### Statistical analysis

Values were expressed as mean + standard deviation (S.D.) for in vitro studies, and as mean + standard error of the mean (S.E.M.) for in vivo studies. One way analysis of variance (ANOVA) followed by post-hoc Dunnett’s multiple comparison test was used to compare the MSC (or IL-6) groups and the control group. The differences between the MSC-CM group and the control group were compared by unpaired *t* test. Statistical analyses were conducted using GraphPad Prism 5.0 software package (GraphPad Software Inc., CA, USA). Differences were considered to be statistically significant when* P* < 0.05.

## Results

### Characterization of morphology, MSC-specific surface markers and multi-differentiation capabilities of hUCMSCs and hAMSCs

**Fig. 1:**
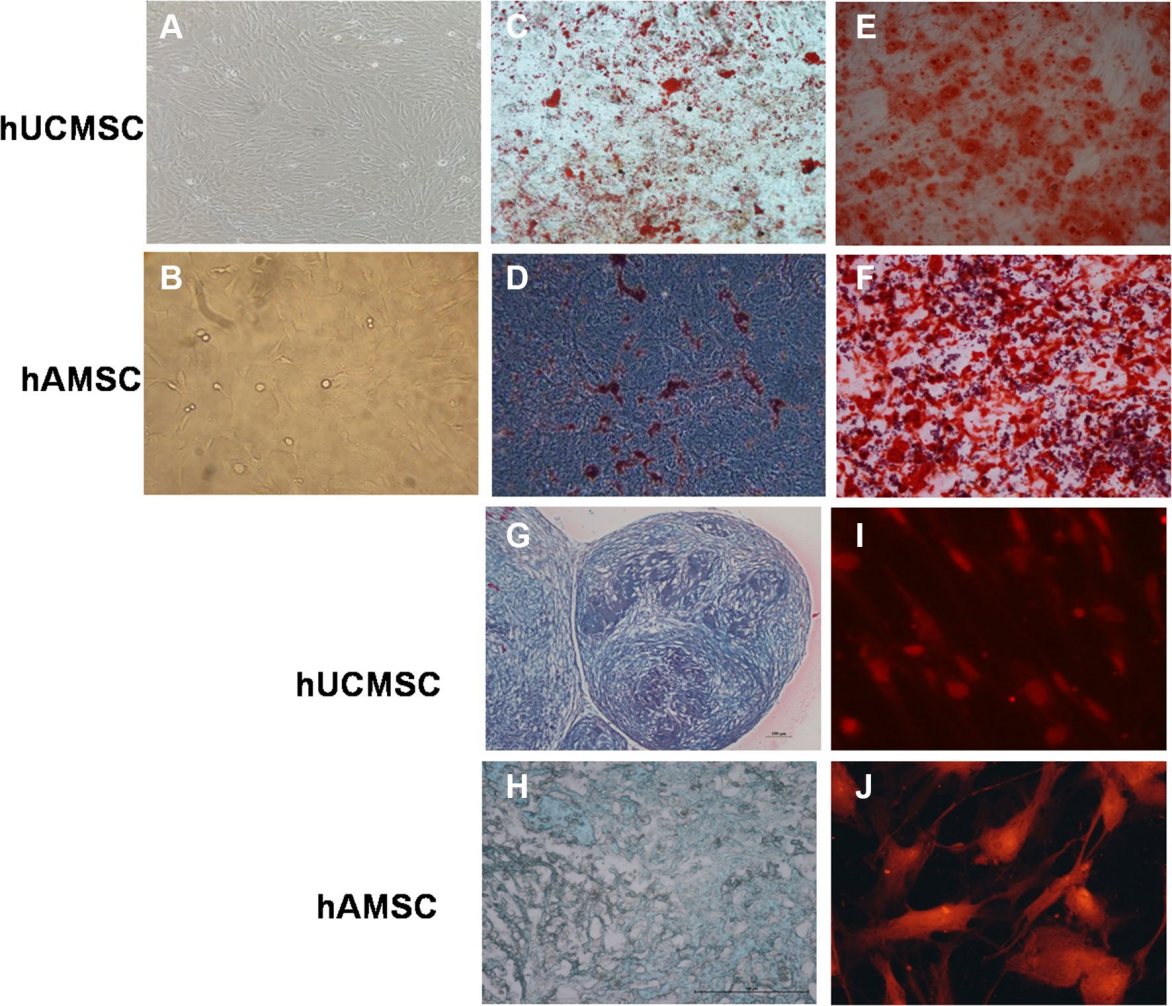
The characteristics of MSCs derived from human umbilical cord and amniotic mesenchymes. The morphology of hUCMSCs (**a**) and hAMSCs (**b**).** c**,** d** hUCMSCs and hAMSCs differentiated into adipocytes. The presence of triglycerides, characteristic of adipocytes, was revealed by staining with oil red O.** e**,** f** hUCMSCs and hAMSCs differentiated into osteoblasts. Calcium deposition was stained with Alizarin Red stain, indicating they were osteoblasts.** g**,** h** Chondrocytes differentiated from MSCs were evaluated by Alcian Blue Staining, which showed the characteristic morphology of cartilage lacuna and sulfated proteoglycan.** i**,** j** Neurogenic differentiation was detected by neurofilament M immunofluorescence staining

The morphology of hUCMSC and hAMSC were basically similar, displaying consistent spindle shape with abundant cytoplasm and large nucleoli. When cells reached 70–80% confluence, they arranged in a whirlpool-like pattern (Fig. 1a, b). The specific surface receptor molecule expression was analyzed by flow cytometry. hUCMSCs and hAMSCs were positive for CD73, CD29, CD14, CD166, CD117, CD49, CD90, CD44, but expressions of HLA-DR, CD45 and CD34 were low. The data showed that the phenotypes of hUCMSCs and hAMSCs were basically indistinguishable from each other (Table 1). Representative photos of MSCs after differentiation are shown in Fig. 1c–j. Under different induction conditions, hUCMSCs and hAMSCs were able to differentiate into adipocytes (Fig. 1c, d), osteoblasts (Fig. 1e, f), chondrocytes (Fig. 1g, h) and neuron-like cells efficiently. The neuron-like cells showed positive expression of neurofilament M, which indicated that MSCs could differentiate into neural cells (Fig. 1i, j).

**Table 1:** The immunophenotypes of hUCMSCs and hAMSCs was analyzed by flow cytometry to study the expressions of specific surface receptor molecules

Specific surface markers	hUCMSC (%)	hAMSC (%)
CD73	91.9	99.4
CD29	99.9	100.0
CD14	99.9	100.0
CD166	98	99.8
CD117	96.9	96.5
CD49	99.8	100
CD90	100.0	99.9
CD44	92.3	99.9
HLA-DR	0.992	0.0882
CD45	0.123	0.281
CD34	2.05	0.289

Data were given as percentage of cells

### The effect of AMSC-CM and UCMSC-CM on proliferation of cancer cells SPC-A-1 and BGC-823

**Fig. 2:**
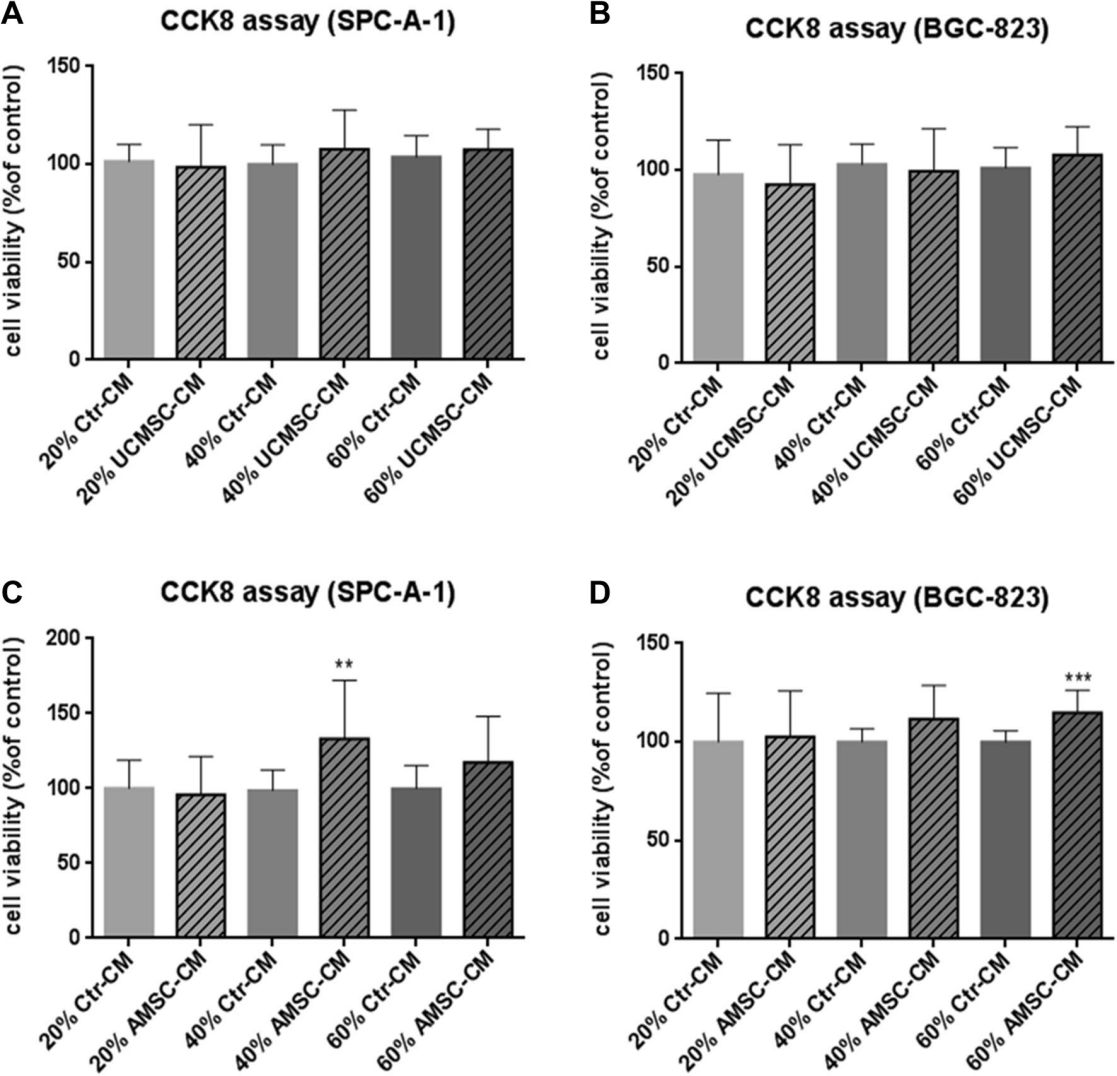
Evaluation of the effect of conditioned medium from MSC (MSC-CM) on the proliferation of cancer cells SPC-A-1 or BGC-823. MSC-CM was incubated for 24 h after seeding the cells in 96-well plates. Cell viability of SPC-A-1 (**a**,** c**) and BGC-823 (**b**,** d**) was assessed by the CCK8 assay after 72 h. Results were expressed as percentages of CCK8 absorbance with respect to the untreated control wells (mean ± SD of three independent experiments with five wells each). Differences between MSC-CM treated groups and control group were determined by Student’s unpaired *t* test. **p < 0.01, ***p < 0.001 comparing MSC-CM treated groups with control

The effect of MSC-conditioned medium on the proliferative activity of malignant cells of different types of cancer including lung adenocarcinoma (SPC-A-1) and gastric carcinoma (BGC-823) was studied. The proliferation of cancer cells was not significantly changed after incubating with all concentration of UCMSC-CM, compared with the control (Fig. 2a, b). The proliferation of SPC-A-1 and BGC-823 was significantly increased after incubating with 40% (*p* < 0.01) and 60% (*p* < 0.001) AMSC-CM, respectively.

### The effect of AMSC-CM and UCMSC-CM on migration of cancer cells SPC-A-1 and BGC-823

**Fig. 3:**
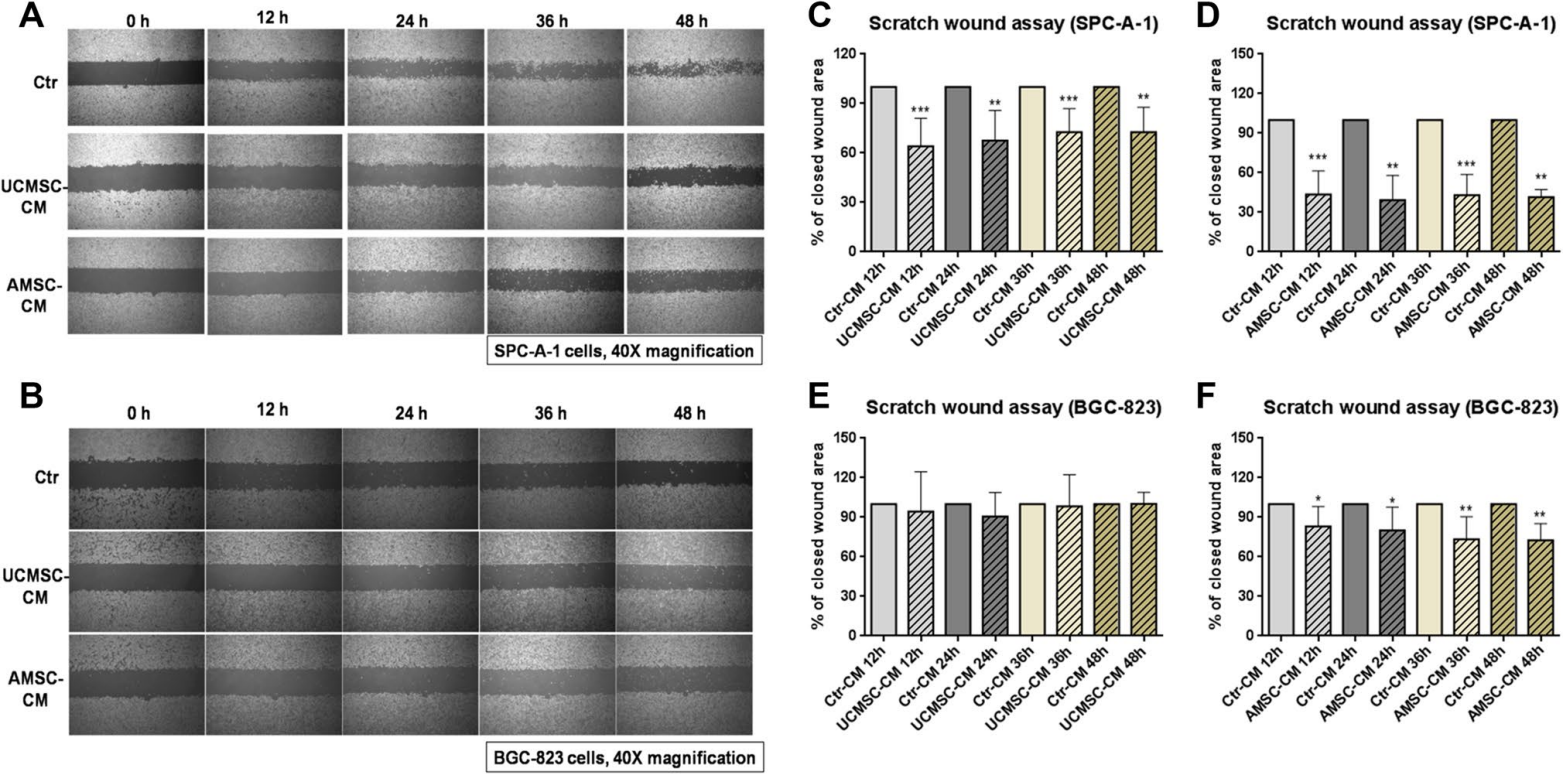
The anti-migratory effect of MSC-CM on SPC-A-1 and BGC-823 cells. The representative photos showing human cancer cells SPC-A-1 or BGC-823 migrating across the scratch wound in the presence or absence of MSC-CM for 12, 24, 36 or 48 h incubation. The results are expressed as the percentage of closed wound area mean + SD of three independent experiments. Differences between MSC-CM treated groups and control group were determined by Student’s unpaired *t* test. *p < 0.05, **p < 0.01, ***p < 0.001 comparing MSC-CM treated groups with control

**Fig. 4:**
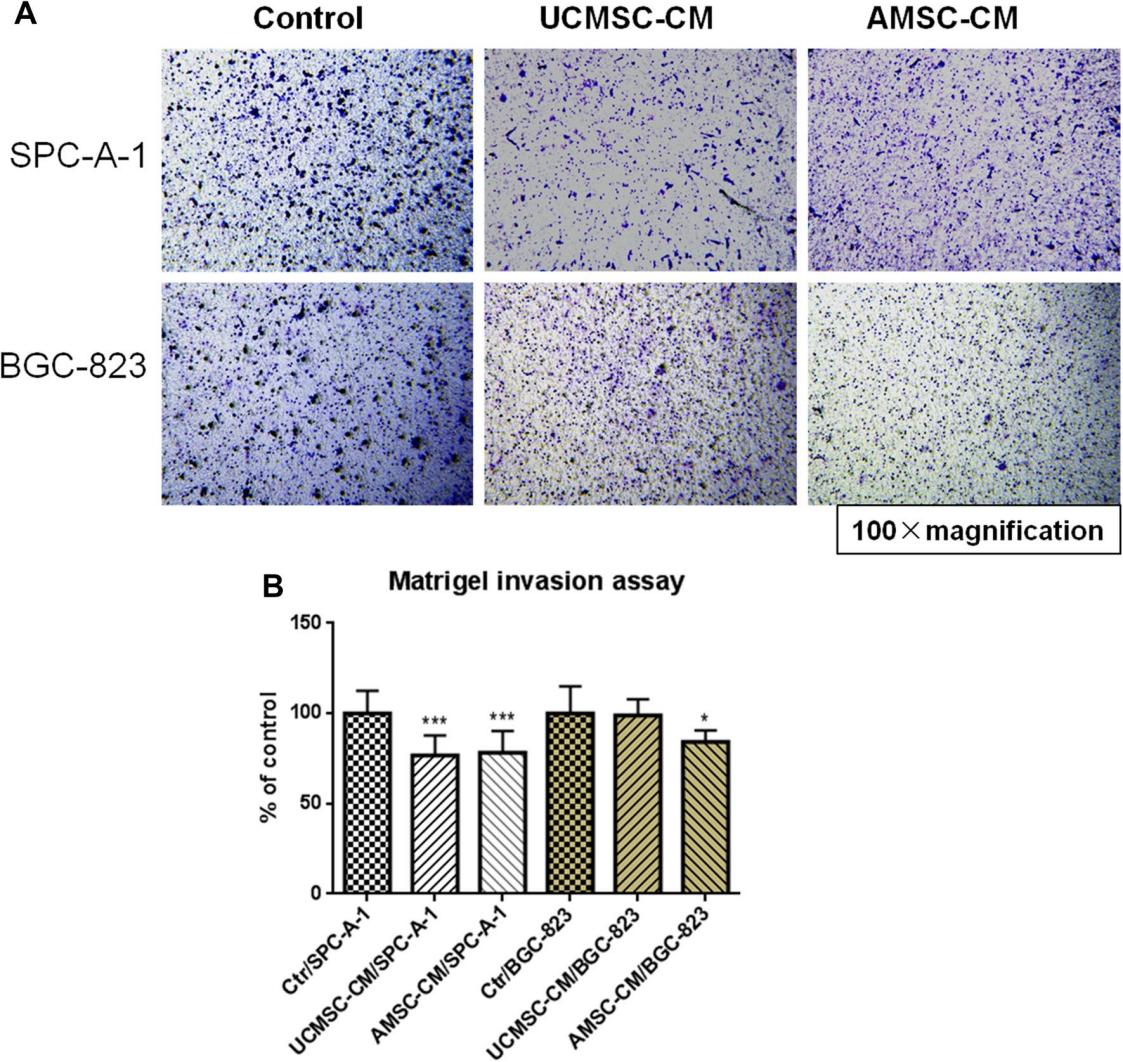
Effects of MSC-CM on migration of SPC-A-1 or BGC-823 cells in Boyden chambers. **a** Representative photomicrographs of the stained cells on the lower side of the membrane. **b** Quantification of migration of SPC-A-1 or BGC-823 cells. Results are expressed as the mean percentage of control (mean + SD of three independent experiments with two wells each). Difference between UCMSC-CM treated group (or AMSC-CM group) and control group with the same cell type was determined by Student’s unpaired *t* test, *p < 0.05, ***p < 0.001 comparing treated groups with control

Migration and invasion of cancer cells is an essential process in cancer metastasis (Guan 2015; Valastyan and Weinberg 2011). The role of MSC-CM on the migration of cancer cells was assessed by the scratch wound assay and Matrigel invasion assay. As shown in Fig. 3a, c, d, incubation with UCMSC-CM or AMSC-CM resulted in decreased closed wound areas in SPC-A-1 cells compared with the respective controls, indicating that both UCMSC-CM and AMSC-CM (*p* < 0.01, *p* < 0.001) had significant inhibitory effects on migration of this type of cells. The motility of BGC-823 cells was also decreased by incubation with AMSC-CM (*p* < 0.05, *p* < 0.01). However, the motility of BGC-823 cells was not affected by incubation with UCMSC-CM (Fig. 3b, e, f). In addition, the results from Matrigel invasion assay showed that lots of cancer cells in the control wells, either SPC-A-1 or BGC-823, migrated from the upper to the lower chamber through the transwell membrane after 24 h incubation (Fig. 4a). In the presence of UCMSC-CM or AMSC-CM, the cell invasion of SPC-A-1significantly reduced by 23.14 ± 10.95% and 21.73 ± 12.00% respectively. Meanwhile, after exposure to AMSC-CM, cell invasion of BGC-823 also reduced by 15.74 ± 6.36%. However, the cell migration ability of BGC-823 incubated with UCMSC-CM was not changed (Fig. 4a, b).

### The effect of AMSC-CM and UCMSC-CM on tube formation of HUVEC

**Fig. 5:**
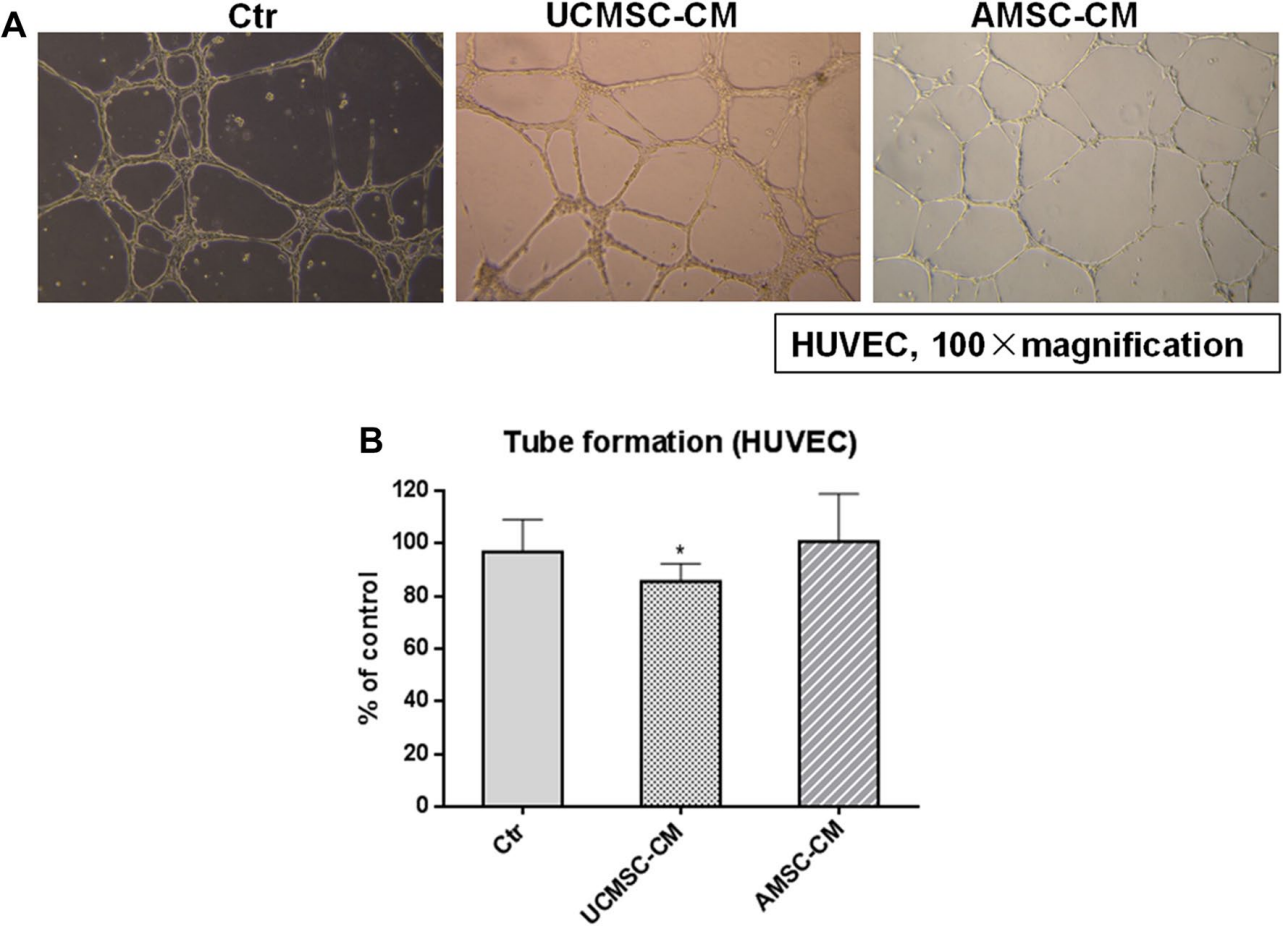
Effects of MSC-CM on capillary-like tube formation by HUVEC. **a** Representative photos showing the tube structures of HUVEC following 8 h MSC-CM incubation. **b** Quantification of tube formation in the presence or absence of UCMSC-CM and AMSC-CM in HUVEC are shown. Results are expressed as the mean percentage of control (mean + SD of three independent experiments). Differences between the MSC-CM treated and untreated control groups were determined by One way analysis of variance (ANOVA) followed by post-hoc Dunnett’s multiple comparisons test, *p < 0.05 as compared to the control group

To investigate whether MSC-CM affected the ability of HUVEC to form capillary-like tubes, Matrigel angiogenesis assay was performed. The tube structures were visible in wells coated with BD Matrigel after 8 h (Fig. 5a). Figure 5b showed that treatment with UCMSC-CM inhibited tube formation of endothelial cells (*p* < 0.05). The effect of AMSC-CM on tube formation of HUVEC was not significant.

### The influence of MSCs co-cultured with cancer cells on cytokines production

Since UCMSC-CM and AMSC-CM could significantly affect the proliferation and migration of SPC-A-1 cells, the release of cytokines from SPC-A-1 co-cultured with UC-MSCs or A-MSCs were simultaneously determined by cytokine antibody array that included 120 cytokines. Among them, 11 inflammatory factors (G-CSF, GM-CSF, ICAM-1, IL-1a, IL-1b, IL-1ra, IL-8, TIMP-2, IL-10, IL-16 and IL-6sR), 8 growth factors (BMP4, PDGF-AA, PDGF-BB, VEGF, EGFR, IGFBP2, IGFBP3 and bFGF) and 11 chemokines (CXCL6, CXCL16, IL-9, IL-18 BPa, LIF, Lymphotactin, MDC, MIF, MIP-3a, GRO and SDF-1a) were remarkably changed after co-culturing, which means these factors were involved in the regulation of MSC on SPC-A-1 cells (Table 2). By comparing to SPC-A-1 or MSC single cultures, the release of 24 cytokines were markedly increased by the co-culturing. Meanwhile, the release of six cytokines was declined after co-culturing. Amongst the down-regulated cytokines, the reduction of VEGF, PDGF and IL-6sR were the strongest. The levels of other cytokines such as bFGF, G-CSF, GM-CSF, ICAM-1, IL-18 BPa, IL-1b, IL-1ra, IL-16 etc., were elevated. The original blots of the cytokine arrays are shown in Supplementary Fig. 3 (standards not shown).

**Table 2:** The levels of different cytokines (growth factors, inflammatory factors and chemokines) from the cultured medium

	SPC-A-1	hAMSC	hUCMSC	hAMSC + SPC-A-1	hUCMSC + SPC-A-1	Down-regulation↓/up-regulation↑
Growth factor (pg/mL)						
BMP4	0.8112 ± 0.1056	7.623 ± 0.5451	0	0	0	**↓**
PDGF-AA	4677.0 ± 0.6645	685.7 ± 3.577	0	157.6 ± 0.1276	2392.0 ± 77.26	**↓**
PDGF-BB	228.9 ± 7.010	0	0	4.206 ± 0.07311	51.79 ± 2.141	**↓**
VEGF	3196.0 ± 62.09	1849.0 ± 7.934	2145.0 ± 4.641	3012.0 ± 84.54	0	**↓**
EGFR	91.10 ± 0.9733	226.4 ± 2.852	26.81 ± 0.1262	585.8 ± 3.592	203.7 ± 4.732	↑
IGFBP2	25.01 ± 1.970	330.5 ± 3.386	1831 ± 26.94	328.4 ± 1.540	179.0 ± 2.674	↑
IGFBP3	12,471.0 ± 358.1	25,699.0 ± 296.7	45,206.0 ± 83.36	39,904.0 ± 24.15	52,154.0 ± 126.6	↑
bFGF	29.59 ± 1.489	99.03 ± 2.029	0	23700.0 ± 306.5	10,729.0 ± 38.19	↑
Inflammatory factors (pg/mL)						
G-CSF	0	4724.0 ± 363.1	202.9 ± 9.382	9641.0 ± 20.72	17,590.0 ± 127.8	↑
GM-CSF	1.512 ± 0.1401	74.34 ± 2.366	1.312 ± 0.07672	345.5 ± 8.865	55.10 ± 2.144	↑
ICAM-1	382.8 ± 6.000	11,608.0 ± 708.4	960.1 ± 44.58	103,220.0 ± 1075.0	32,104.0 ± 1373.0	↑
IL-1a	0.5851 ± 0.04151	13.16 ± 1.032	0.7068 ± 0.007003	526.3 ± 2.877	128.9 ± 2.420	↑
IL-1b	0	4.449 ± 0.1699	0	333.9 ± 6.337	76.58 ± 4.972	↑
IL-1ra	0	11.66 ± 1.795	0	14.97 ± 0.7150	48.80 ± 0.2888	↑
IL-8	37.95 ± 4.429	561.3 ± 41.03	392.7 ± 3.617	564.6 ± 30.07	846.1 ± 57.21	↑
TIMP-2	21,603.0 ± 384.0	33,886.0 ± 777.5	28,915.0 ± 956.5	41,663.0 ± 674.3	57,251.0 ± 3522	↑
IL-10	0.2297 ± 0.02256	0.01087 ± 0.01538	0	1.011 ± 0.1266	0.6640 ± 0.09464	↑
IL-16	0	0	0	75.70 ± 3.796	62.74 ± 13.09	↑
IL-6sR	108.2 ± 1.653	0.04951 ± 0.07001	13.15 ± 0.2290	0.4488 ± 0.03371	1.522 ± 0.3130	**↓**
Chemokines (pg/mL)						
CXCL6	0	170,666.0 ± 3506.0	4.265 ± 0.7100	318,080.0 ± 12988.0	1,857,356.0 ± 105486.0	↑
CXCL16	9034.0 ± 107.7	181.4 ± 7.893	352.6 ± 36.76	973.0 ± 59.85	5929.0 ± 179.7	**↓**
IL-9	0	0	0	1405.0 ± 362.1	557.1 ± 8.800	↑
IL-18 BPa	0	0	0	250.8 ± 31.01	129.4 ± 28.80	↑
LIF	0	105.6 ± 9.569	0	19,546.0 ± 17.60	2941.0 ± 12.78	↑
Lymphotactin	0	0	0	17.61 ± 4.622	11.01 ± 1.031	↑
MDC	0	0.5591 ± 0.1456	13.95 ± 1.700	121.6 ± 21.64	310.7 ± 68.18	↑
MIF	29.40 ± 0.2155	56.30 ± 3.544	29.57 ± 4.763	414.7 ± 52.37	306.2 ± 22.25	↑
MIP-3a	211.2 ± 5.169	8050.0 ± 341.0	142.4 ± 10.15	14362.0 ± 1123	13619.0 ± 775.3	↑
GRO	12.54 ± 1.858	880.0 ± 14.62	826.0 ± 75.97	1209.0 ± 271.4	1879.0 ± 215.3	↑
SDF-1a	0.1409 ± 0.1752	0	2.952 ± 0.8212	3.789 ± 0.8587	5.237 ± 0.4809	↑

### The effects of hAMSCs and hUCMSCs on tumor formation and promotion in vivo

**Fig. 6:**
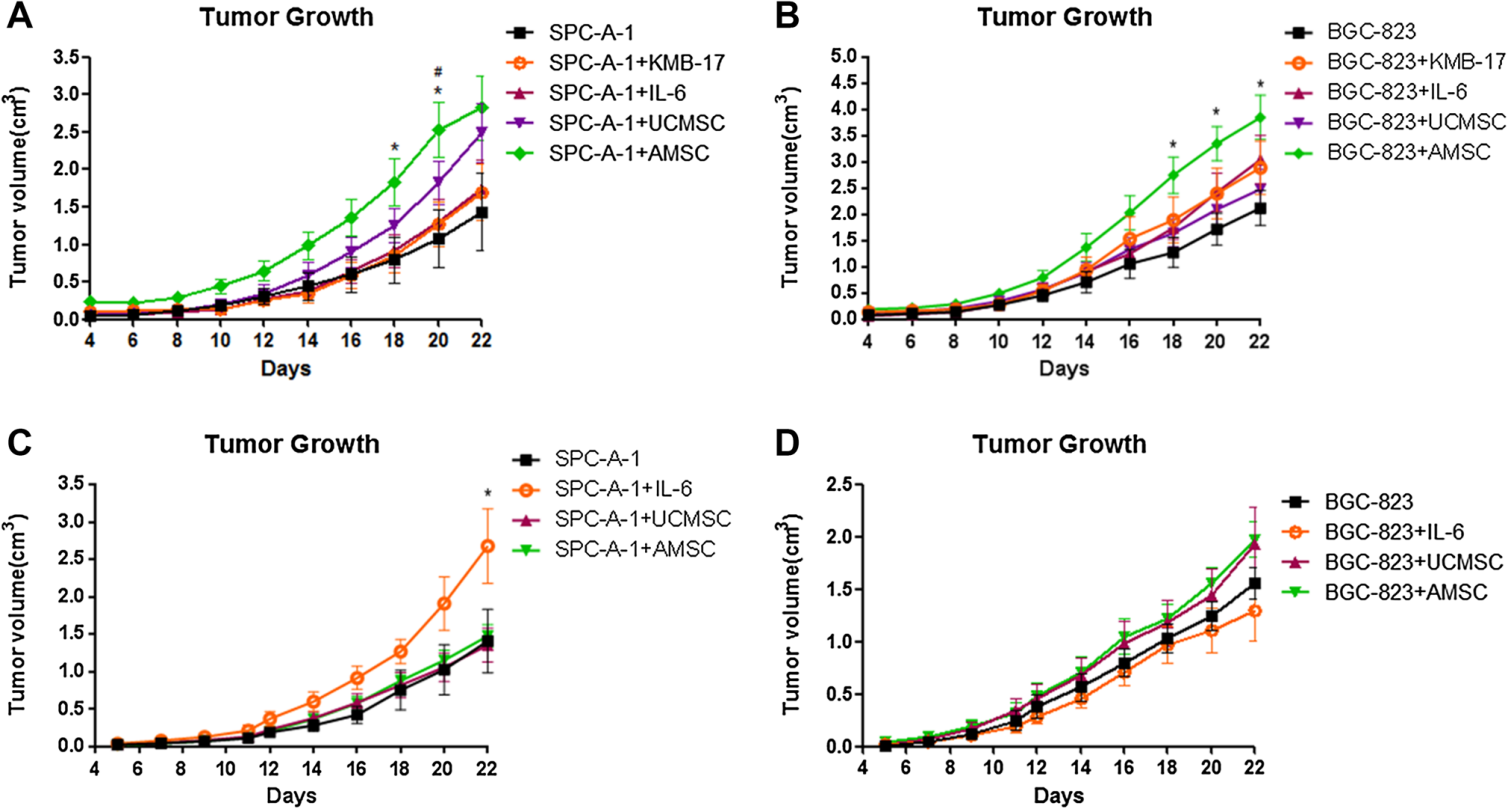
Effects of MSCs on nude mice model of xenograft tumor.** a**,** b** Mice were co-injected subcutaneously with SPC-A-1 or BGC-823 cancer cells and MSCs (hUCMSCs or hAMSCs). The cancer cells with KMB-17 was used as control in this animal model. *p < 0.05 SPC-A-1 + AMSC vs. SPC-A-1, ^#^*p* < 0.05 SPC-A-1 + AMSC vs. SPC-A-1 + KMB-17, *p < 0.05 BGC-823 + AMSC vs. BGC-823 (one-way ANOVA followed by post-hoc Dunnett’s multiple comparison test). Each point represented a mean + S.E.M. of 6 or 7 tumors.** c**,** d** Mice were injected subcutaneously with SPC-A-1 or BGC-823 cancer cells. The following i.v. injection of MSCs (hUCMSCs or hAMSCs) commenced on day 12, *n* = 6–7. IL-6 was used as the positive control in this animal model. *p < 0.05 SPC-A-1 + IL-6 vs. SPC-A-1 (one-way ANOVA followed by *post-hoc* Dunnett’s multiple comparison test)

To further evaluate the effects of MSC on tumor promotion in vivo, two animal models based on subcutaneous xenografts were employed in our study. Tumor xenografts were established successfully in nude mice after subcutaneous injection with both SPC-A-1 and BGC-823 cells. From the co-injection experiment, the tumor size of the SPC-A-1/hAMSCs group was significantly increased compared with SPC-A-1/KMB-17 or SPC-A-1 alone (*p* < 0.05, Fig. 6a). Meanwhile, the tumor size of BGC-823/hAMSCs group was also larger than those of the BGC-823-alone group (*p* < 0.05, Fig. 6b). These findings indicated that AMSCs were likely to be able to promote tumor growth, which was consistent with our in vitro result from CCK8 assay. On the other hand, no significant differences in tumor volume were observed in groups injected with SPC-A-1 or BGC-823 alone with the respective hUCMSCs co-injection groups, and SPC-A-1 or BGC-823 alone with the respective KMB-17 co-injection groups (Fig. 6a, b).

For another set of animal experiment, hUCMSCs or hAMSCs (or IL-6 as the positive control in this model) was intravenously injected into the mice through tail veins on the 12th day after tumor inoculation, when the tumors nodules were observed. As shown in Fig. 6c, the tumor of SPC-A-1 cells grew faster after treatment with IL-6 (*p* < 0.05), which was consistent with previous studies (Saglam et al. 2015). However, this effect was not observed in the co-injection experiment animal model (Fig. 6a, b). Notably, no significant difference in tumor volume was found among the SPC-A-1 (or BGC-823) with or without hUCMSCs (or hAMSCs) suggesting that MSCs did not promote tumor growth in this animal model (Fig. 6c, d).

To confirm the effect of MSC on tumor growth through cell proliferation, a Ki-67 immunostaining assay was performed on mice tumor sections. When compared with the other groups, the number of Ki-67 positive cells in tumor samples from SPC-A-1 or BGC-823 with hAMSCs was higher (Supplementary Fig. 1A&B). Moreover, in i.v. animal model, the cells with Ki-67 positive in the tumor regions from the IL-6-treated group (inoculated by SPC-A-1) were increased when compared with those from other groups, including SPC-A-1 alone, BGC-823 alone, and the respective hUCMSCs or hAMSCs co-injection groups (Supplementary Fig. 2A).

### The effects of hAMSCs and hUCMSCs on cancer metastasis in vivo

**Fig. 7:**
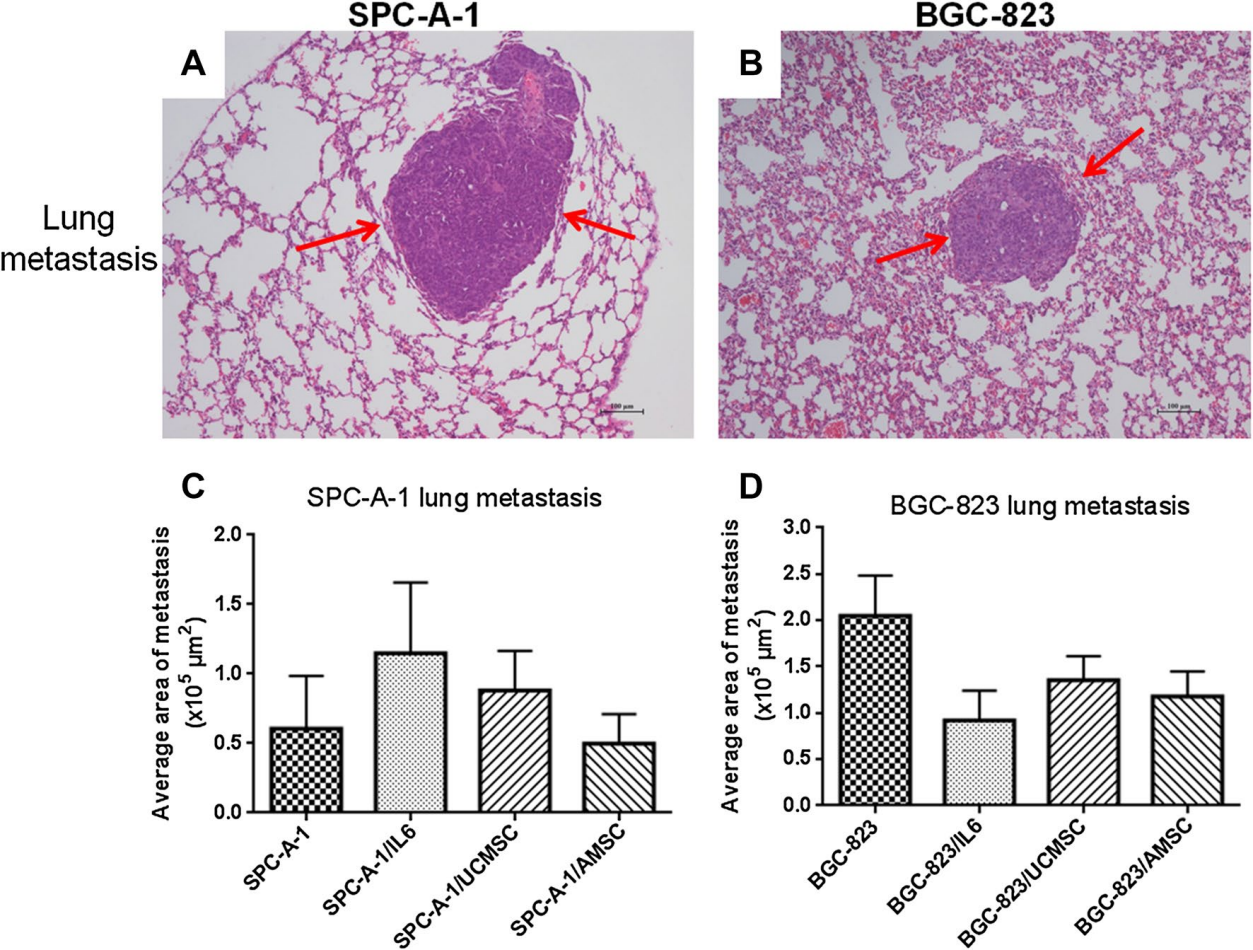
Effects of MSCs on the murine experimental metastatic model. H & E staining was used to evaluate the lung metastasis of SPC-A-1 (**a**,** c**) or BGC-823 (**b**,** d**).** a**,** b** The representative photos of micro-metastasis in lungs with red arrows showing the tumor cells.** c**,** d** The histograms represented the tumor lesions in lungs as assessed by histological analysis, and expressed as an average tumor area per group in absolute units (µm^2^). Data shown in mean + SEM, *n* = 6–7 in each group

No tumor metastatic lesions in other organs such as liver and lungs were observed in murine subcutaneous xenograft cancer model (Data not shown). To evaluate the ability of MSCs to induce tumor metastasis development, the murine experimental metastatic model was used. From our data, micro-metastasis of SPC-A-1 and BGC-823 in lungs were observed after H&E staining, as shown in red arrows in Fig. 7a, b. However, there were rarely obvious tumor metastatic lesions in the liver or bone, even in the murine experimental metastatic model. As shown in Fig. 7c, there were neither inhibitory nor induction effects of hAMSCs or hUCMSCs on tumor metastatic lesions in SPC-A-1 cancer model. At the same time, the lung metastasis in groups treated with IL-6 was slight increased, which was consistent with the tumor promotion effect of IL-6 in SPC-A-1 subcutaneous xenograft cancer model (Fig. 6c). The BGC-823 groups treated with hUCMSCs, hAMSCs or IL-6 did not show significant promotion in metastasis comparing with the control group (Fig. 7d).

## Discussion

MSCs from different tissues presented diverse effects on cancer progress (Akimoto et al. 2013). Due to the fact that MSCs showed different properties under malignant conditions, they need to be further investigated thoroughly and chosen carefully to balance the efficacy and safety for any particular cancer type. Although some studies showed that the MSCs from bone marrow promoted cancers such as osteosarcoma, colorectal tumor and gastric cancer (Tsai et al. 2011; Tu et al. 2012; Xue et al. 2015), the MSCs from adipose tissue or umbilical cord showed inhibitory effects on tumor cells from prostate tumor and glioma (Cavarretta et al. 2010; Ma et al. 2014). Obviously, the phenomenon was not absolute (Akimoto et al. 2013). The conflicting results regarding the pro- and anti-tumorigenic effects of MSCs on cancer may be due to the varying ratios of MSCs–cancer cells being administered, the different immune responses from immunocompromised or immunocompetent murine hosts, the diverse oncogenes, mutations, receptors, dysfunctioning of pathways on different kinds of cancer cell lines, and so on (Meng et al. 2018; O’Malley et al. 2016). In this study, MSCs from two different sources, umbilical cord and amniotic membrane, were simultaneously established to evaluate the effects on two different cancer cells SPC-A-1 and BGC-823, which were lung adenocarcinoma and gastric carcinoma respectively. The results demonstrated that MSCs from the umbilical cord was safer than those from the amniotic membrane, because UCMSCs not only did not promote the proliferation on the cancer cell lines and the ability of tube formation of human umbilical vein endothelial cells in vitro, but also inhibited the migration of cancer cells. Although AMSCs also inhibited the migration of cancer cells, the proliferation was indeed enhanced.

MSCs together with cancer cells (SPC-A-1 or BGC-823) were transplanted subcutaneously into BALB/c nude mice to observe the effects of MSCs on tumor growth in an in vivo model. In particular, the interactions of MSCs with other cell types, including tumor cells, immune cells, cancer-associated fibroblasts, endothelial cells of blood and lymphatic vessels, could modulate tumor development (Feng and Chen 2009). Recent studies showed that MSCs exerted their immunomodulatory effects, both immune-suppressive and promotive, in many pathological condition (Knaan-Shanzer 2014). Thus, the use of immunodeficient animal model might ignore the interaction among the cancer cells, host immune cells and MSCs. Xenograft models using immune competent animal models should be considered to resolve this problem. Each of the two animal models in the present study had its own advantages. The co-injection of MSCs with cancer cells into the mice best mimics the direct contact of the two types of cells. However, the intravenous injection of MSCs belonged to systematic infusion. MSCs appeared to migrate to tumor sites and played the roles through paracrine signals in the tissue microenvironment. This was closer to a clinical setting (Belmar-Lopez et al. 2013). Moreover, two different tumor xenografts from two cancer types, namely gastric carcinoma and lung adenocarcinoma, were employed to provide more representative information. Our in vivo results showed that AMSCs promoted tumor growth in co-injection with both SPC-A-1 and BGC-236 xenografts. This result was not observed in intravenous injection animal model. On the other hand, UCMSCs did not increase tumor size and Ki-67 expression in tumor sections in both animal models. No obvious side effects were observed after intravenous administration of hAMSCs and hUCMSCs in mice. Some studies demonstrated that IL-6 could promote tumor growth (Tu et al. 2012). Thus, IL-6 was used as the positive control in in vivo studies. However, the promotional effect of IL-6 was not obvious in co-injection model, which might be associated with the route of administration. For the intravenous injection model, IL-6 only showed promotional effects on SPC-A-1 xenografts, indicating its regulatory activity was cell-specific. Based on the current results, hUCMSCs did not have carcinogenic or cancer-promoting activities, which was more suitable for clinical use as comparing with hAMSCs. In addition, our previous study had evaluated the carcinogenic ability of UCMSCs in experimental animals and the normal chromosome karyotype of hUCMSCs (passages 7–23) (Meng et al. 2018), further supporting its potential use in clinical applications.

For assessment of the effects of MSCs on cancer metastasis, the murine experimental metastatic model was better than subcutaneous xenograft cancer model because there was rarely metastatic lesion in the latter. Metastasis is a complicated progress including tumor growth, angiogenesis, epithelial–mesenchymal transition, homing, and so on. The murine experimental metastatic model just mimicked the later progresses of cancer cell metastasis such as survival in the blood circulation, metastatic colonization and organ tropism (Quail and Joyce 2013). When cancer cells were injected via the tail vein, they first arrived at the lungs through the circulatory system. Therefore, the tumor metastatic lesions in our murine experimental metastatic model were mainly located in the lungs rather than liver or bones, which was a limitation of this model. Our results showed that MSCs did not significantly enhance the lung metastasis of SPC-A-1 or BGC-823, which was consistent with the in vitro data.

MSCs could secrete cytokines through paracrine pattern that participate in the regulation of different signaling pathways for cell proliferation, differentiation, migration, etc. (Knaan-Shanzer 2014) hAMSCs and hUCMSCs had been shown to inhibit the migration of cancer cells by regulating cytokine production in vitro. However, the lack of understanding of the mechanical and chemical interactions of the transplanted MSCs with the factors present in the cancer cells limited their use in cancer treatment. For the first time, our study conducted systematic analysis on cytokines from SPC-A-1 co-cultured with both UCMSCs and AMSCs. The results showed that BMP-4, PDGF-AA, PDGF-BB, VEGF and CXCL16 decreased after co-culturing, which might be associated with the anti-migration and anti-angiogenesis effects of MSCs (Deng et al. 2010; Feng and Chen 2009; Kim et al. 2015). However, there were still many cancer promoting factors being increased, such as EGFR, G-CSF, CXCL6, etc. (Nicholson et al. 2001; Verbeke et al. 2011). In general, administration with MSCs showed the ability of equilibrating the multiple factors in the microenvironment of cancer cells that led to tumor growth in vivo. Although the immunophenotypes of hUCMSCs and hAMSCs were basically similar (Table 1), the cytokines from the two MSCs, either co-cultured with cancer cells or not co-cultured, were greatly distinctive (Table 2). That may explain the difference in effects of hUCMSCs and hAMSCs on cancer cells. The role of MSCs in the pathogenesis and development of cancer is sophisticated and likely associated with the balance of competing inhibition and promotion forces. Due to the complex regulatory network, the detailed mechanisms need to be further studied based on our findings.

Taken together, the present study evaluated the effects of hUCMSCs and hAMSCs on cancer cell in vitro and in vivo. From our results, hUCMSCs did not affect the proliferation of cancer cells in both SPC-A-1 and BGC-823 cell lines, and inhibited the migration of SPC-A-1 as well as the tube formation of epithelial cells. Moreover, this was the first report that obtained the comprehensive cytokine secretion profile of human UCMSCs or AMSCs co-cultured with SPC-A-1 cancer cells, which provided the molecular basis for further studies. In vivo studies also found that hUCMSCs did not promote tumor development (tumor proliferation as well as lung metastasis). Therefore, the present findings provided scientific evidence on the safety and advantage of the use of hUCMSCs in pre-clinical setting.

## Electronic supplementary material

Below is the link to the electronic supplementary material.

Supplementary material 1 (DOCX 3599 KB)
