# Supplementary material for: Assessment of tumor promoting effects of amniotic and umbilical cord mesenchymal stem cells in vitro and in vivo
Source: J Cancer Res Clin Oncol. 2019 Feb 25;145(5):1133–46. doi: 10.1007/s00432-019-02859-6 (PMC6482126; doi:10.1007/s00432-019-02859-6)
Supplement: Supplementary file 1 — Supplementary material 1 (DOCX 3599 KB) [file 432_2019_2859_MOESM1_ESM.docx]

Supplementary Figure 1

Figure S1. Ki67 immunostaining analysis of co-injection of MSC with cancer cells' effects on tissue sections from the tumor. Six samples of each group were examined and the images were randomly taken from four visual fields per sample.

Supplementary Figure 2

Figure S2. Ki67 immunostaining analysis of MSC effects on tissue sections from the tumor. MSC was intravenously injected into xenograft bearing nude mice through tail veins. Six samples of each group were examined and the images were randomly taken from four visual fields per sample.

Supplementary Figure 3


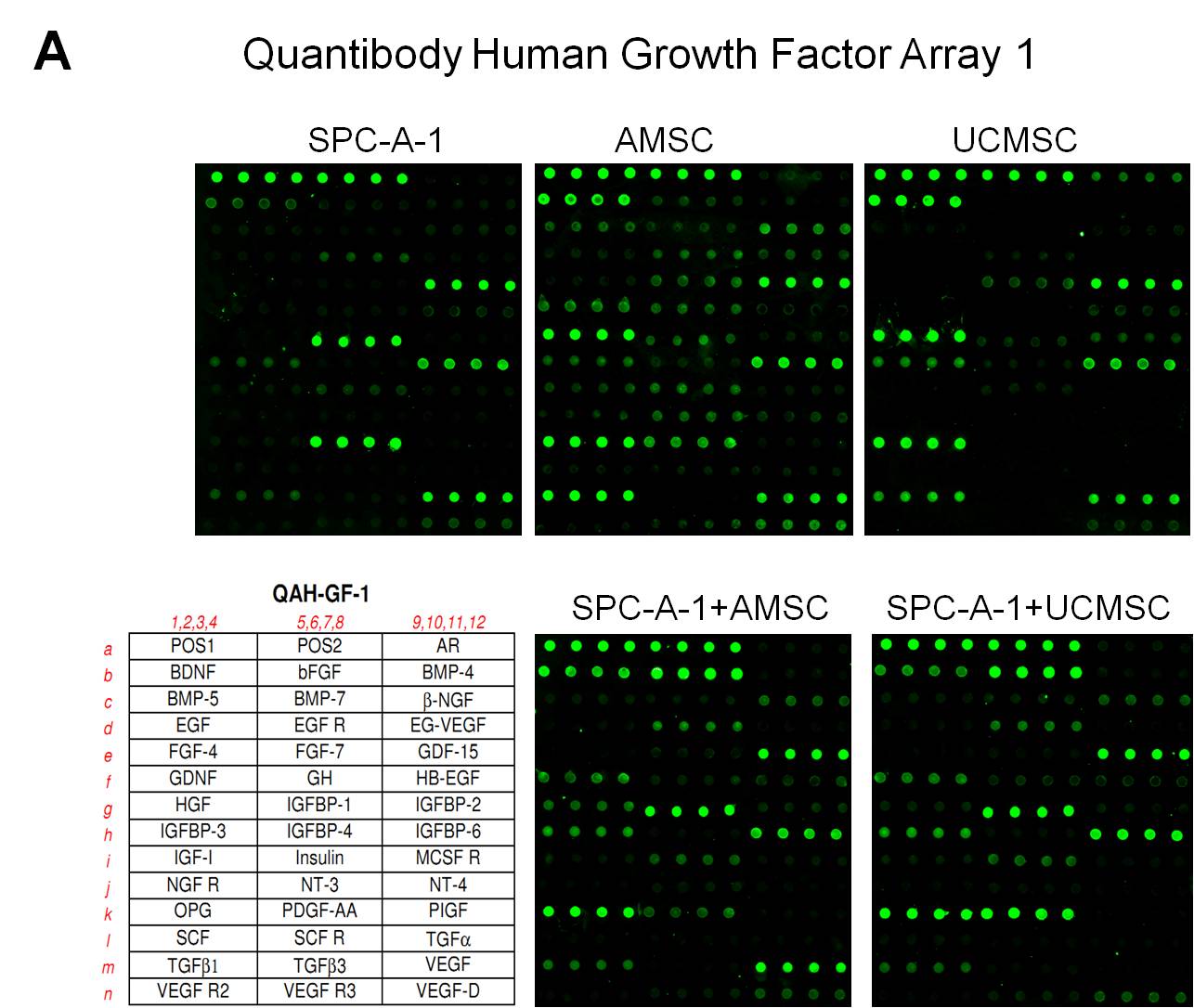


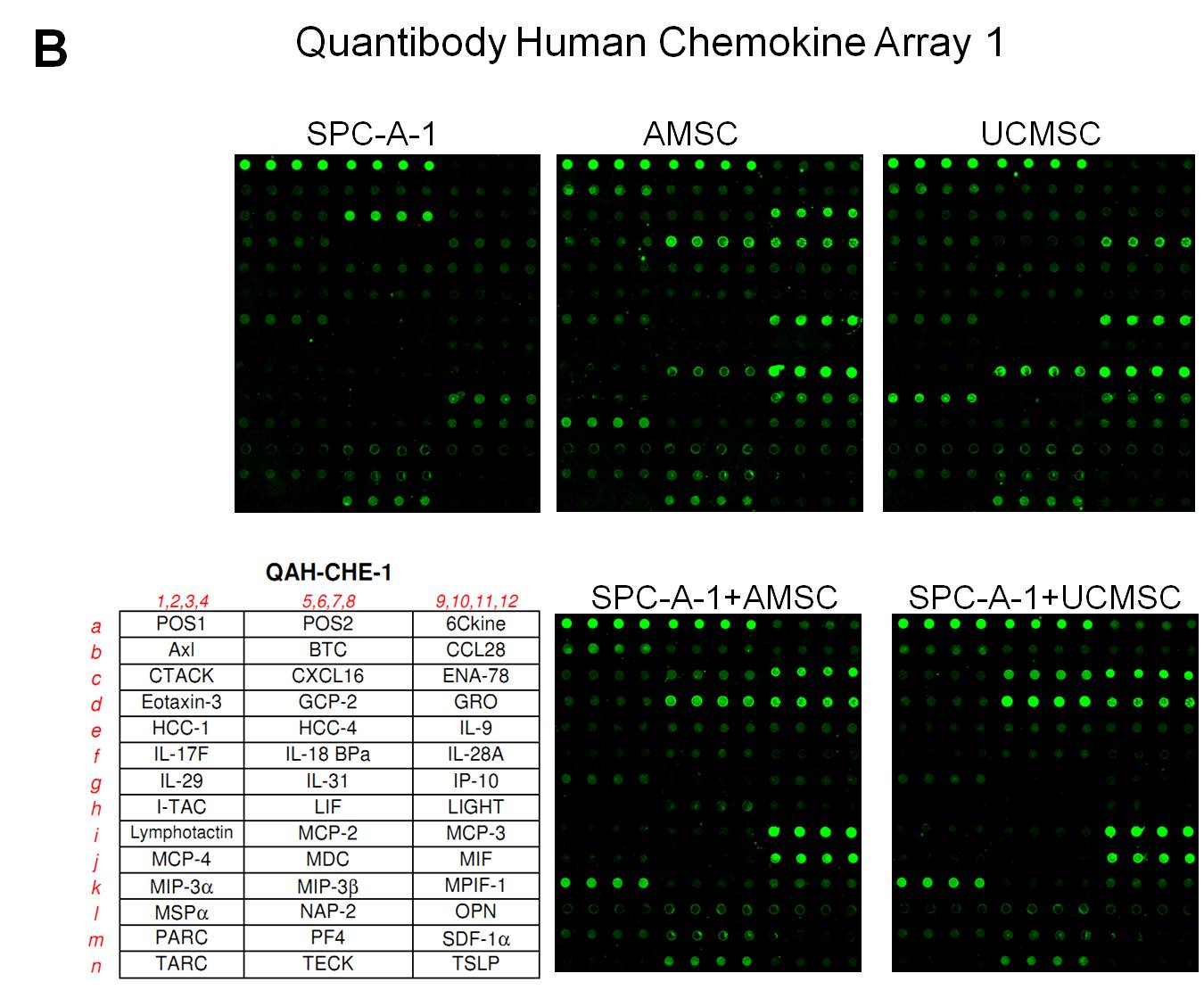


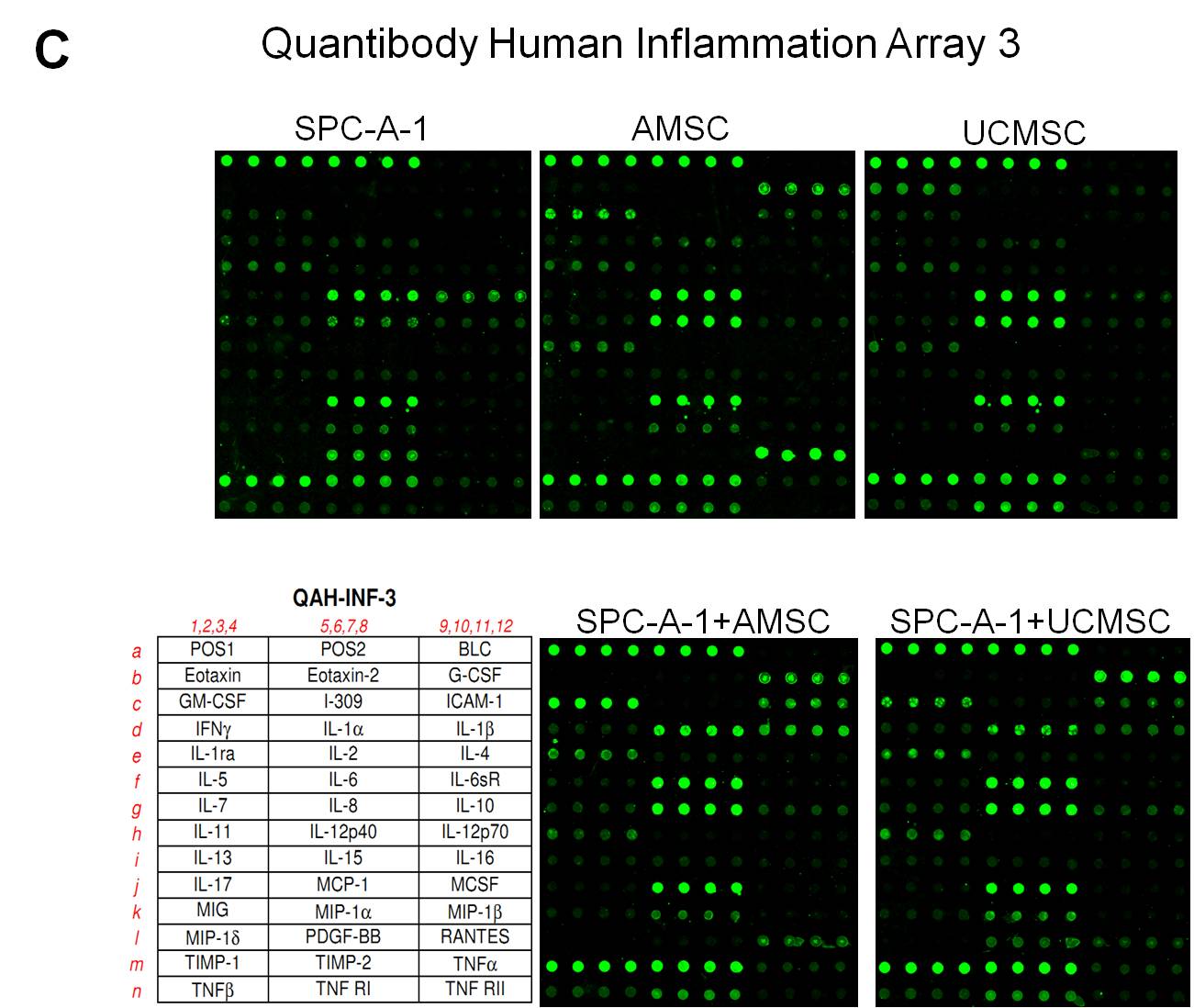


Figure S3. The original blots of the cytokine arrays for 40 human growth factors (A), 40 human chemokines (B), and 40 human inflammatory factors (C).
